# Complication during transportation and 30 days mortality of patients with acute coronary syndrome

**DOI:** 10.1186/s12872-020-01327-1

**Published:** 2020-01-17

**Authors:** Michał Czapla, Dorota Zyśko, Tom Quinn, Piotr Karniej

**Affiliations:** 1 Department of Organisation and Management, Faculty of Health Sciences, Wroclaw Medical University, Bartla 5, 51-618, Wroclaw, Poland; 2 Department of Emergency Medicine, Faculty of Health Sciences, Wroclaw Medical University, Wroclaw, Poland; 3 Faculty of Health, Social Care and Education Kingston University and St George’s, University of London, London, UK

**Keywords:** Sudden cardiac arrest, Acute coronary syndrome, Emergency medicine, Medical transport, Prehospital emergency care

## Abstract

**Background:**

Patients with acute coronary syndrome (ACS) who present to hospitals without interventional facilities frequently require transfer to another hospital equipped with a cardiac catheterization laboratory. This retrospective cohort study evaluates the association of the type of medical transport with patient outcomes.

**Methods:**

A retrospective analysis of medical records of patients with ACS transported by basic (BT) and specialist transfer (ST) by emergency medical teams (EMTs). We analyzed age, gender, hemodynamic parameters, type of the emergency medical team, and complications during transport as well as patient survival to hospital admission, survival time and the 30-day mortality rate.

**Results:**

Of 500 patients who underwent transfer, ST transported 368 (73.6%) and BT 132 (26.4%) patients (*p* < 0.001). Complications during transportation occurred in 3 (1%) in the ST group and 2 (1.5%) in and BT group. Cardiac arrest during transfer occurred in no (0%) patients in the ST group, and 2 (1.5%) in the BT group (*p* = 0.118). Survival to admission was recorded in all patients in the ST group and 131/132 (0.8%) patients in the BT group (*p* = 0.592). 40 (12%) of patients in the ST group and 13 (11%) patients in the BT group (*p* = 0.731) died within 30 days of transfer.

**Conclusions:**

Complications during medical transport of ACS patients from hospitals without a cardiac catheter lab to hospitals equipped with such a lab were rare and their incidence was not associated with the type of transporting EMT. The type of EMT was not associated with 30-day patient mortality.

## Background

Acute coronary syndromes (ACS) are common and carry significant short and longer term risks to the patient. For patients with ST-segment elevation myocardial infarction (STEMI) guidelines recommend timely access to a hospital capable of performing percutaneous coronary intervention (PCI-capable hospital). For patients presenting with non-STEMI ACS, guidelines recommend a primary PCI strategy in cases of haemodynamic instability or shock, refractory ischaemic pain, mechanical complications or recurrent dynamic ST-segment or T-wave changes [1].

The onset of ACS symptoms prompts help-seeking behavior by patients. Depending on the severity of the symptoms, their characteristics and the assessment of the threat to health or life made by the patient and/or their family, the patient has the following options: calling an ambulance, ordering a house call by a physician or nurse, seeing the primary care physician on their own, going to a hospital admissions room or a hospital emergency department (ED) [2].

Given that in Poland the patient has several options regarding how to contact the health care facilities, it is not surprising that such contact is frequently made from outside a hospital equipped with an interventional cardiology (catheterization) unit. In Poland, just over half (56%) STEMI patients are admitted directly to a PCI-capable hospital [2]. Therefore, a substantial number of patients who present initially to a non-PCI capable hospital require transportation by an emergency medical team (EMT) to the nearest PCI-capable hospital [3–5].

The organization of health care for ACS patients is an important factor influencing chances of survival [6]. Reduction of time between the first medical contact and the performance of coronary angiography and angioplasty if indicated is a key recommendation of international guidelines [1].

In Poland, the national emergency medical services (EMS) provide front-line emergency response, but do not undertake interhospital transfers, which are the responsibility of private ambulance providers contracted by individual hospitals. These latter emergency medical teams (EMTs) are organized in two forms: a) a basic team (BT) consisting of at least two persons authorized to carry out medical emergency procedures (paramedics, most of whom receive their professional qualifications following a higher education diploma, and emergency nurses with secondary medical or higher nursing education, holding specialist qualifications in the field of emergency nursing); b) a specialist team (ST) comprising at least three persons, one of whom is always a physician. It is, therefore, practicable to identify differences in procedures and the association of both types of teams with regard to ACS patients. Considering the differences between paramedics, nurses and doctors as regards education, training, skill level and authority (e.g. in relation to advanced airway management and some medicines), we hypothesise that paramedics and emergency nurses in the Polish emergency medical system provide safe and effective care of patients, comparable to that provided by physician-led specialist teams during interhospital transfer of ACS patients.

Therefore, the objective of this study was to assess whether the type of team (basic or specialist) transporting the ACS patient was associated with patient survival to admission at the PCI-capable hospital and on the 30-day mortality.

## Methods

### Study design and setting

This study comprised a retrospective analysis of medical records of 500 patients with ACS transported since 1st January 2010 to 31st August 2015 by specialist and basic EMTs belonging to the Polish Emergency Medical Services company in Wroclaw (Poland), from admission rooms, hospital emergency departments and other departments of 7 hospitals without PCI capability in the Lower Silesian region of Poland, to PCI-capable hospitals. No patient with ACS was excluded.

### Study population

In all the studied cases it was possible to transport the patient both by a specialist team (with a physician) and by a basic team (manned by paramedics), and the decision regarding the type of transport was made by a physician employed in the hospital who issued the transport order. No formal written protocols for ordering transportation by an EMT were available at the time of the study, other than that the EMT should arrive within 30 min of physician request.

### Ethical considerations

This study was approved by the independent Bioethics Committee of the Wroclaw Medical University (decision no. KB–513/2016). All participants were asked to gave their informed consent to participate in this study. The study was carried out in accordance with the tenets of the Declaration of Helsinki and reccommendations of Good Clinical Practice.

### Statistical analyses

Statistical analysis was performed using the Statistica 12 (TIBICO Inc., USA) software under licence of the Wroclaw Medical University, Poland. Patients were divided into two groups, depending on the type of the transporting EMT, i.e. the specialist transport group (ST) and the basic transport group (BT). For continuous variables, the arithmetic means and standard deviations were calculated and then tested with the Shapiro-Wilk test to determine the type of distribution. For qualitative variables, we calculated the frequency of their occurrence. Continuous variables were compared using the parametric t-Student test for independent trials or the nonparametric Mann-Whitney test, depending on the fulfillment of test assumptions. The chi-square test was used to compare qualitative variables. Logistic regression analysis and backward stepwise regression analysis of the dependence of 30-day survival was performed based on independent variables such as age, gender, hemodynamic parameters, applied treatment and type of EMTs, which carried out the order of medical transport. The results were considered statistically significant if the *p*-value is *p* < 0.05.

## Results

### Characteristics of study population

The studied group comprised 500 patients: 292 (58.4%) men and 208 (41.6%) women (*p* < 0.001). Mean age in the study population was 68.7 ± 13.9 years. Female patients had a mean age of 72.6 ± 12.3, significantly higher than the age of male patients, which was 66.0 ± 14.3 years (*p* = 0.018). Baseline clinical characteristics are shown in Table 1. We were unable to identify ACS phenotype (STEMI, non-STEMI) from the documentation provided.

**Table 1:** Demographic characteristics and hemodynamic parameter of patients in the group transported by specialist and basic EMT

	Group ST (*n* = 368)	Group BT (*n* = 132)	*P*-value
Age (years)	68.1 ± 14.4 Min-Max: 19.0–93.0	70.5 ± 12.3 Min-Max: 37.0–93.0	0.098
Sex n (%)	Female - 150 (41%) Male - 218 (59%)	Female - 58 (44%) Male - 74 (56%)	0.525
SBP	133.9 ± 24.6	132.5 ± 22.5	0.590
DBP	78.8 ± 13.8	78.4 ± 14.7	0.791
HR	84.2 ± 19.2	86.2 ± 22.5	0.330

*Abbreviations: ST* Specialist emergency medical team, *BT* Basic emergency medical team, *n* Number of patients, *Min* Minimum value, *Max* Maximum value, *SBP* Systolic blood pressure, *DBP* Diastolic blood pressure, *HR* Heart rate

### Emergency medical teams

A group of 132 persons (26.4%) were transported by a basic EMT (the BT group) and 368 (73.6%) persons by a specialist EMT (the ST group) (*p* < 0.001). Comparing the ST and BT groups, no significant differences were found with regard to patients’ age and gender distribution. There were no statistically significantly differences in baseline hemodynamics parameters between the groups. Baseline demographic and hemodynamic data are provided in Table 1.

### Complications during medical transport

Complications occurred during transportation in 1% of patients in the ST group and 1.5% of patients in the BT group (*p* = 0.366). Two patients (0.4%) in the BT group suffered cardiac arrest during transport. One patient transported by ST required mechanical ventilation. Cardiac arrest occurred in no patients in the ST group (0%) and two in the BT group (1.5%) (*p* = 0.118).

In one case of cardiac arrest during transport, the presenting rhythm was pulseless electric activity (PEA) and in the other case asystole. In the first case the patient was successfully resuscitated, in the second case the patient died. Single cases of other complications were also reported. One patient transported by a specialist EMT had respiratory failure during transport (the mechanically-ventilated patient), one person had recurrent ventricular tachycardia treated with intravenous amiodarone, and one patient in cardiogenic shock was treated with catecholamines (administration started in ambulance). All three patients survived to hospital admission and to 30 days.

### Survival to hospital admission

Survival to hospital admission was recorded in 499 patients (99.8%); 368 patients in the ST group and 131 patients in the BT group.

### Thirty-day mortality

30-day mortality data were obtained in 453 patients, 333 in the ST group and 120 in the BT group. 40 patients (12%) in the ST group and 13 patients (11%) in the BT group (*p* = 0.731) died within 30 days of transfer (Table 2). Patients who died within the 30-day period were older (73.7 ± 11.1 vs. 60.8 ± 14.1), had lower SBP (119.1 ± 24.1 vs. 135.1 ± 23.4) and DBP (72 ± 14.8 vs. 79.2 ± 14.3) values and a significantly lower SatO2 (93.4 ± 6.4 vs. 96.2 ± 3.5) at baseline.

**Table 2:** Demographic and haemodynamic parameters in groups of patients who died within 30 days of transport to the cardiac catheterization lab and who survived this period

	Patients survived beyond 30 days (*n* = 400)	Patients died within 30 days (*n* = 53)	*P*-value
Male sex n (%)	230 (57.5)	32 (60.4)	0.690
Age (years)	60.8 ± 14.1	73.7 ± 11.1	0.010
SBP	135.1 ± 23.4	119.1 ± 24.1	< 0.001
DBP	79.2 ± 14.3	72 ± 14.8	< 0.001
HR	84.7 ± 29.1	87.7 ± 22.2	0.350
SatO_2_	96.2 ± 3.5	93.4 ± 6.4	< 0.001

*Abbreviations: SBP* Systolic blood pressure, *DBP* Diastolic blood pressure, *HR* Heart rate, *SatO*_*2*_ Saturation, *N* number of patients

Based on the data obtained in the study, we created models for logistic regression analysis. The dependent variable was survival of the ACS patient beyond 30 days since the day of medical transport. The independent variables were: age, gender, the occurrence of SBP < 90 mmHg, SBP 140–179 mmHg, SBP ≥180 mm, HR < 55/min, HR > 90/min, administration of catecholamines, use of oxygen therapy, a recorded SatO2 ≥ 92% without administration of oxygen or ≥ 95% in patients who received oxygen, as well as the type of EMT. The results of the logistic regression and backward stepwise regression analysies for 30-day survival are shown in Table 3.

**Table 3:** Logistic regression and backward stepwise regression analyses: dependent variable death within 30 days since the day of medical transport to the cardiac catheterization lab

	Regression analysies		Backward stepwise regression	*P*-value
	OR (95% CI)	*P*-value	OR (95% CI)	*P*-value
Transport by basic EMT	0.76 (0.37–1.55)	0.447		
Transport from ED/AMU	0.63 (0.31–1.25)	0.186		
Female sex	0.41 (0.16–1.02)	0.055		
Age (for a year)	1.04 (1.01–1.07)	0.018	1.03 (1.01–1.06)	0.012
SBP ≥180 mmHg	0.89 (0.10–7.51)	0.911		
SBP < 90 mmHg	3.58 (0.74–17.34)	0.113		
SBP 140–179 mmHg	0.40 (0.18–0.91)	0.028	0.40 (0.20–0.83)	0.014
HR < 55/min	2.28 (0.43–11.93)	0.330		
HR > 90/min	1.51 (0.74–3.11)	0.259		
SatO_2_ < 90%	0.63 (0.17–2.40)	0.502		
SatO_2_ ≥ 92% without oxygen therapy or ≥ 95% with oxygen therapy	0.20 (0.08–0.51)	0.001	0.20 (0.10–0.39)	< 0.001
Administration of catecholamines	2.31 (0.32–16.53)	0.403		

*Abbreviations: EMT* Emergency medical team, *ED* Mergency department, *AMU* Acute medical unit, *SBP* Systolic blood pressure, *DBP* Diastolic blood pressure, *HR* Heart rate, *SatO*_*2*_ Saturation, *OR* Odds ratio

The full-model logistic regression analysis showed that factors significantly correlated with a lower risk of death were: age (the younger the patient, the lower the risk of death), SBP in the range of 140–179 mmHg, SatO2 ≥ 92% without oxygen therapy or ≥ 95% when treated with oxygen therapy.

**Fig. 1:**
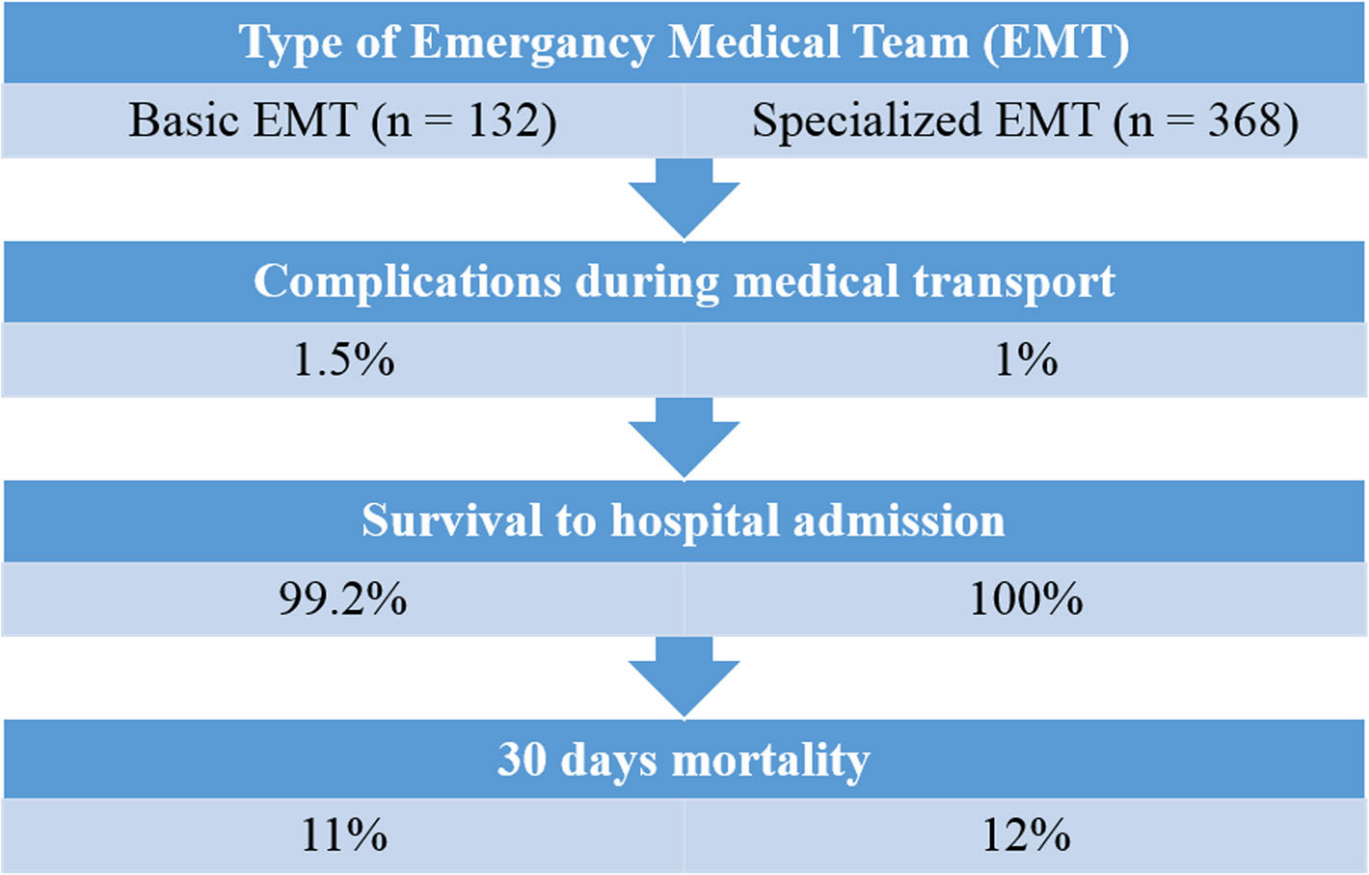
A summary of the main findings of the study

In the backward stepwise logistic regression analysis, only three variables were significantly correlated with death within 30 days. These were age (the older the patient, the higher the risk of death), SBP < 90 mmHg and a failure to obtain the SatO2 ≥ 95% with oxygen therapy or ≥ 92% in cases where the patient did not receive oxygen. Figure 1 presents a summary of the main findings of the study.

## Discussion

The need to transport patients from a non-PCI-capable hospital to a PCI-capable hospital is an important issue, when almost half of STEMI patients (and many with non-STEMI) present at first instance to a non-PCI capable facility. Stachowiak et al. [7] demonstrated that 16% of patients were transported to a PCI-capable hospital by a basic EMT, 25% by a specialist EMT, and as many as 59% of the patients first reported either to a non PCI capable hospital, to a primary care physician or to a private cardiologist’s practice, from where they needed to be transported to a PCI-capable hospital. Difficulties in comparing the findings of the present study with results of studies carried out in other countries stem, among other factors, from heterogenous organization of emergency medical systems. As types of the EMTs vary between countries, the scope of medical intervention falling within the competencies of each EMT is different.

As discussed earlier, Poland operates two types of EMTs: a basic EMT, with paramedics or emergency nurses (specialized in the field of emergency nursing) and a specialist EMT which must always include a physician. In Poland, paramedics are mainly graduates of medical colleges, trained at the level of bachelor’s degree. Likewise, nurses in Poland are currently also trained only in medical colleges. Considering the fact that in some other countries paramedics and nurses are not always university educated, it would be practicable to carry out similar analyses in other countries to facilitate comparative analysis [8].

In Japan, Fujii et al. [9] reported that over 65% of patients were transferred to a hospital by emergency medical services, of which over 13% were taken initially to a non-PCI capable hospital, necessitating subsequent interhospital transfer to a PCI-capable hospital. A quarter (24%) of all patients self-presented at non-PCI capable hospitals and required interhospital transport. In Quebec, Lambert et al. [10] found that of 774 patients, 441 were brought to the first hospital by ambulance, and then 213 were further transported to another, PCI-capable by the same EMT, while 228 patients had to wait 4–12 min for another ambulance transport. Patients who self-presented to the hospital were also transported to another facility. Using the same ambulance for inter-hospital transport reduces the time to arrival at a PCI-capable facility, and is associated with improved outcomes. In Ireland McKee at al [11] reported that out of 1894 patients, 38% initially contacted their primary care physician, while in the United States, Fosbol et al. [12] reported that out of 6010 patients as many as 49% required transport from a non-PCI facility to a PCI- capable hospital.

The above data confirm that irrespective of the country, ACS patients frequently present to non-PCI capable hospitals, subsequently requiring interhospital transfer. This also suggests that inter-hospital transports are a common strategy employed not only in Poland, but also in other countries and on other continents.

### Demographic information

In Poland, the average age at which patients experienced a myocardial infarction leading to hospitalization or death is 63 years for men and 74 years for women. Men constituted 58% of all the patients [13]. Tousek et al. [14] demonstrated that within the context of age at which myocardial infarction occurred, men constituted 63%. Trojanowski et al. [15] reported a higher percentage of male patients presenting with this condition, namely 68%. In common with other countries, in Poland, ACS is more common in men than in women.

### Hemodynamic parameters

Baseline demographic and hemodynamics characteristics in the present study are comparable with reports from other countries [16, 17].

### Complications during transport and rates of survival

Our study found that complications rarely occurred during medical transport irrespective of the type and capabilities of the team undertaking the transfer. Complications in the ST group were recorded in only 1% of patients, and in the BT group in 2% of patients. The proportion of patients who died during transportation was very low. This is in contrast to findings from other studies. Fosbol et al. [18] reported that pre-hospital cardiac arrest was more frequent in patients who first came to a non-PCI capable hospital than in those who were transported by an EMT to a PCI-capable hospital (9.5% vs. 2.8%). In-hospital mortality was lower in patients transported directly to a PCI-capable hospital 6.3% vs. 9.3%).

The differences between our study findings and those of other authors may be because the patients who were at greatest risk, i.e. presented with shock, severe pain or collapse may have called emergency medical services immediately, had a pre-hospital ECG performed and been transported directly to a PCI-capable hospital. A study conducted in Paris on 8181 patients with ACS showed that patients with acute, short-term symptoms who call an ambulance were at a greater risk of death than those with less acute symptoms [19]. These observations may account for the low complication and mortality rate in our study.

### 30-day survival

In the United Arab Emirates Callahan et al. [20] reported 30-day mortality in patients transferred from a non-PCI capable hospital to a PCI-capable facility at 30 days, 1/128 (0.8%) patients transported between hospitals by emergency medical services had died. In USA, Al-Zaiti et al. [21] reported that where ambulance services transported STEMI patients from their home to a hospital, the 30 day mortality was 11%. In turn, Thang et al. [22] demonstrated that in ACS patients transported to a hospital by ambulance, the 30-day mortality was 4.3%. A lower mortality was also reported by May et al. [16], where the 30-day mortality for such transports amounted to 2.9%. Based on the above studies conducted in different countries, we may conclude that the 30-day mortality rate varies and depends on the studied population. It must be noted that in the studies by Thang et al. [22] and May et al. [16], the patients were transported directly to a PCI capable facility. In our study we were unable to assess the time needed for the patient to reach a hospital equipped with an interventional cardiology lab, although some patients waited for transfer in a hospital located as far as 30 km away from the destination PCI-capable hospital. Another important aspect is that these patients always waited for the arrival of another transport team. Given the time dependent nature of ACS – particularly STEMI – we may assume that such delays could adversely impact the patients’ chances of a good outcome.

In our study, the logistic regression analysis showed that the patient’s chances of survival were reduced by 4% with each year of age and increased by 60% when the SBP was 140–179 mmHg compared to the SBP < 90 mmHg, and also increased by 80% in the case of normal levels of saturation as compared to reduced saturation. The backward stepwise logistic regression analysis demonstrated a fivefold higher risk of death for patients with SPB < 90 mmHg. The risk of death increased with age (the older the patient, the higher the risk of death) and increased by 73% in cases where the saturation levels of ≥95% with oxygen therapy and ≥ 92% without oxygen therapy were not obtained.

Schoos et al. [23] found that, among others, the patient’s age, pulse > 100/min, diabetes and chronic kidney disease were all statistically significantly correlated with the risk of death within 30 days. The risk of death increased nearly threefold if the heart rate was > 100/min. Studies by other authors [24–27] confirm that the patient’s age affects the 30-day mortality.

### Limitations

Our study has several limitations. Firstly, we did not have access to referring physicians’ decision making regarding type of transportation team requested. Secondly, the records we assessed did not include potentially informative variables such as ACS phenotype, time from symptom onset, biomarker assays, Killip class or past medical history such as diabetes or hypertension. Thirdly, we were unable to ascertain how patients first presented to the non-PCI capable hospital, of what treatments had been administered prior to transfer. Fourthly, we did not have access to information on treatments given at the PCI-capable hospital (e.g. received intervention or medication) which may have influenced outcomes. We recommend future studies collect a more comprehensive data set for analysis.

## Conclusions

Complications during medical transport of ACS patients from non-PCI capable hospitals to PCI-capable hospitals in our study were rare and their incidence was not associated with the type of the transporting EMT. The type of the EMT transporting the patient was not associated with 30-day survival. ACS patients can be transported by basic EMT and specialized EMT are only necessary for high-risk patients. Paramedics and emergency nurses in the Polish emergency medical system provide safe and effective care of patients, comparable to that provided by physician-led specialist teams during interhospital transfer of ACS patients.

## Data Availability

The datasets used and/or analyzed during the current study are available from the corresponding author on reasonable request.

## Abbreviations

ACS: Acute coronary syndrome
BT: Basic transfer
DBP: Diastolic blood pressure
ECG: Electrocardiogram
ED: Emergency department
EMS: Emergency medical service
EMT: Emergency medical team
PCI: Percutaneous coronary intervention
PEA: Pulseless electric activity
SBP: Systolic blood pressure
ST: Pecialist transfer
STEMI: ST-segment elevation myocardial infarction
